# Decline of gastric cancer mortality in common variable immunodeficiency in the years 2018-2022

**DOI:** 10.3389/fimmu.2023.1231242

**Published:** 2023-10-06

**Authors:** Cinzia Milito, Federica Pulvirenti, Giulia Garzi, Eleonora Sculco, Francesco Cinetto, Davide Firinu, Gianluca Lagnese, Alessandra Punziano, Claudia Discardi, Giulia Costanzo, Carla Felice, Giuseppe Spadaro, Simona Ferrari, Isabella Quinti

**Affiliations:** ^1^ Department of Molecular Medicine, Sapienza University of Rome, Rome, Italy; ^2^ Reference Center for Primary Immune Deficiencies, AOU Policlinico Umberto I, Rome, Italy; ^3^ Rare Diseases Referral Center, Internal Medicine 1, Ca’ Foncello Hospital, AULSS2 Marca Trevigiana, Department of Medicine (DIMED), University of Padova, Padova, Italy; ^4^ Department of Medical Sciences and Public Health, University of Cagliari, Cagliari, Italy; ^5^ Department of Translational Medical Sciences, University of Naples Federico II, Naples, Italy; ^6^ Medical Genetics Unit, IRCCS Azienda Ospedaliero-Universitaria di Bologna, Bologna, Italy

**Keywords:** gastric cancer, common variable immunodeficiency, mortality, endoscopy, screening, Helicobacter pylori, gastric adenocarcinoma, COVID-19

## Abstract

**Introduction:**

In patients with Common Variable Immunodeficiency, malignancy has been reported as the leading cause of death in adults, with a high risk of B-cell lymphomas and gastric cancer.

**Methods:**

We conducted a five-year prospective study aiming to update the incidence and mortality of gastric cancer and the incidence of gastric precancerous lesions in 512 CVID patients who underwent a total of 400 upper gastrointestinal endoscopies.

**Results:**

In the pre-pandemic period, 0.58 endoscopies were performed per patient/year and in the COVID-19 period, 0.39 endoscopies were performed per patient/year. Histology revealed areas with precancerous lesions in about a third of patients. Patients who had more than one gastroscopy during the study period were more likely to have precancerous lesions. Two patients received a diagnosis of gastric cancer in the absence of Helicobacter pylori infection. The overall prevalence of Helicobacter pylori infection in biopsy specimens was 19.8% and related only to active gastritis. Among patients who had repeated gastroscopies, about 20% progressed to precancerous lesions, mostly independent of Helicobacter pylori.

**Discussion:**

While gastric cancer accounted for one in five deaths from CVID in our previous survey, no gastric cancer deaths were recorded in the past five years, likely consistent with the decline in stomach cancer mortality observed in the general population. However, during the COVID-19 pandemic, cancer screening has been delayed. Whether such a delay or true decline could be the reason for the lack of gastric cancer detection seen in CVID may become clear in the coming years. Due to the high incidence of precancerous lesions, we cannot rely on observed and predicted trends in gastric cancer mortality and strongly recommend tailored surveillance programs.

## 1 Introduction

In patients with Inborn Errors of Immunity (IEI), high morbidity and mortality associated with non-infectious comorbidities, such as inflammatory diseases and cancers, has been widely described in several cohorts of adult patients (1–4). In a previous study, we demonstrated that among malignancies, gastric adenocarcinoma (GC) was the leading cause of death in a cohort of Italian CVID patients with a 10.1-fold excess mortality and with a cancer onset fifteen years earlier than the normative population (5). The mechanisms of lymphomagenesis and carcinogenesis have been related to chronic antigenic stimulation and defective immune surveillance (6). However, despite the impact of malignancies, up to now international consensus guidelines for early detection in IEI are still lacking. The high morbidity and mortality due to GC have been confirmed by several studies in different geographic areas, showing a standardized incidence ratio (SIR) ranging from 5.7 to 10.3 (7–9). More recently, a higher SIR (16.5) has been observed in a German cohort in the period 2015-2021 (10). Most of the published data came from retrospective analysis, and a better figure might be clear only by performing prospective studies, allowing the development of recommendations on the use of routine upper gastrointestinal (GI) endoscopy to screen GC (11).

Starting in 2018, we planned a prospective five-year cohort study enrolling 512 CVID patients, with the aim of updating the incidence and mortality related to GC and the incidence of precancerous gastric lesions.

## 2 Methods

### 2.1 Study design

We conducted a five-year prospective study in a cohort of 512 adult CVID patients (>18 years old), regularly followed in four University-based Inborn Errors of Immunity (IEI) referral centers located in Central Italy (Rome), Southern Italy (Naples), Northern Italy (Padua-Treviso), and Sardinia Island (Cagliari). To be considered for analysis, subjects needed to fulfill the 2016 ESID/PAGID revised criteria for CVID (https://esid.org/).

Data were prospectively collected from 01/01/2018 to 31/12/2022. The five-year period was divided into two intervals: a) pre–COVID-19 period (January 2018 - December 2019), and b) COVID-19 period (January 2020 - December 2022). During the study period, we collected demographic and clinical data and histological findings from gastric and duodenal biopsies from upper GI endoscopy. For each patient, we collected gender, date of birth, date of CVID diagnosis, data on cancer diagnosis and histology, date of last follow-up, vital status information, date and cause of death, and past medical history of Helicobacter Pylori (HP) infection. For each endoscopy, histological findings, and the presence of HP were collected. Crude mortality ratio for GC was calculated as ratio of number of deaths for GC and the mean number of CVID patients at risk in a five-year period.

The study was approved by the Ethical Committee of the Sapienza University of Rome, Prot. 316/2016, and Prot. 0279/2021, and was performed in accordance with the Good Clinical Practice guidelines, the International Conference on Harmonization guidelines, and the most recent version of the Declaration of Helsinki.

### 2.2 Statistical analysis

Sociodemographic and clinical variables were summarized with descriptive statistics. Continuous variables were described using median and interquartile ranges (IQR) and categorical variables using frequencies and percentages. Group differences were analyzed by the Mann-U Whitney test to compare continuous variables for non-normally distributed data and by Student’s t-test for normally distributed data; the χ2 test was used for categorical variables. A p-value of <0.05 was used to consider differences statistically significant. For mortality analysis, the time since diagnosis was determined using the age at the time of CVID diagnosis. The endpoint used was the time of the last known follow-up or the date of death. Probabilities of survival after the diagnosis of CVID were estimated from the Kaplan-Meier life Table. Statistical analyses were performed with SPSS 18.0 software for Windows (SPSS, Chicago, IL, USA) and GraphPad Prism version 8.0.0 for Windows, GraphPad Software, San Diego, CA USA.

## 3 Results

### 3.1 Patients

In the study period January 2018 - December 2022, 512 subjects with a CVID diagnosis were included in the dataset. Participants’ characteristics (**Table 1**) indicated that most were female (54%, n=273), aged >50 years (48%, n=247), and had received a CVID diagnosis >10 years before (57%, n=280). Sixteen percent of patients (n= 80) received the CVID diagnosis during the study period. Out of 512 CVID patients, 377 have been already enrolled in our previous cohort study (5). The previous and the current surveys enrolled CVID patients with similar ages, male/female ratio, and age at CVID diagnosis (**Supplementary Table 1**).

**Table 1:** Demographic and clinical characteristics of 512 CVID patients enrolled in the study.

	Overall cohort n=512	Patients who underwent upper GI endoscopy n=263
Sex (female), n (%)	273 (54)	150 (57)
Age at enrollment, median (IQR)	49 (36-59)	47 (36-58)
Time from CVID diagnosis (years), median (IQR)	11 (6-18)	11 (6-18)
Age ≥ 50 years, n (%)	247 (48.5)	123 (46.8)
Time from diagnosis ≥10 years	280 (57.1)	171 (64.5)
Age at diagnosis (years), median (IQR)	39 (29-52)	38 (30-50)
Patients diagnosed in the last 5 years, n (%)	80 (16)	38 (15)

CVID, common variable immunodeficiency; GI, gastrointestinal; IQR, interquartile range; n, number.

### 3.2 Upper GI tract endoscopies

During the whole study period a total of 400 upper GI tract endoscopies were performed in 263 patients. At the enrollment, 64% of patients who underwent gastroscopy had CVID for ≥10 years, 21% for ≥5 years and 15% were diagnosed during the study time. Forty-seven percent (n=123) were aged over 50 years.

The main indication for the initial gastroscopy was cancer surveillance screening (55%), with 39% of patients having at least one risk factor for GC (age>50 years, male sex, cigarette smoke, high BMI, precancerous lesions or previous HP infection). Other indications included gastrointestinal symptoms (39%) or portal hypertension follow-up (7%).

**Figure 1:**
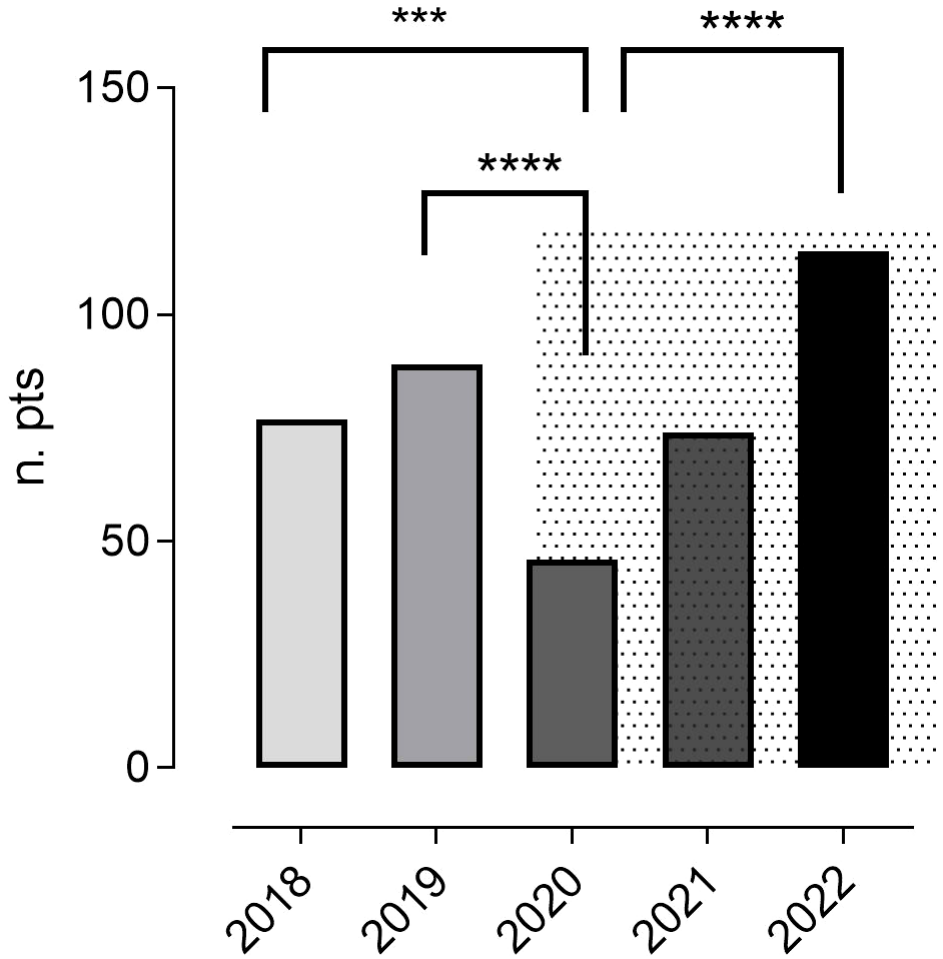
The annual number of patients who underwent ≥ one upper GI tract endoscopy during the study period. The pandemic time is represented by a grey background. In 2020, the first year of the pandemic, the number of patients who performed an upper GI endoscopy was reduced compared to 2018 and 2019. This fall was reversed in 2021 and 2022. Chi-square test was used to evaluate statistical significance. Two-tailed P value significances are shown as: ***p<0.001, ****p<0.0001. n. pts, number of patients.

In the pre-pandemic period (January 2018 - December 2019), a total of 166 upper GI tract endoscopies were done in 142 patients (0.58 per patient/year), and in the COVID-19 period, a total of 234 upper GI tract endoscopies were performed in 197 patients (0.39 per patient/year). In particular, 77 patients underwent upper GI endoscopies in 2018, and 89 in 2019. In 2020, the first year of the pandemic, the number of patients who performed an upper GI endoscopy was 46 (2019 vs 2020, p<0.0001). This fall was partially reversed, rising to 74 in 2021, and to 114 in 2022 (**Figure 1**).

### 3.3 Upper GI tract endoscopies

Gastric histology. Among 263 patients who underwent one or more upper GI tract endoscopies, two patients were diagnosed with GC. Areas with precancerous lesions were identified in 72 (28%), as 17% of patients had intestinal metaplasia (IM) (n=46), 2% had dysplasia (n=6), and 19% (n=50) had atrophic gastritis. Active gastritis was recorded in 51% (n=134) of patients. Histological features of gastric ulcers were not recorded. Moreover, nodular/follicular lymphoid hyperplasia (NH/FH) was found in 29% (n=77). One patient had a diagnosis of gastric HP-positive MALT (**Table 2**).

**Table 2:** Gastric mucosal histology and HP status in 263 CVID patients who performed one or more upper GI tract endoscopies.

	Patients who underwent ≥ one upper GI tract endoscopy									
	Overall n=263		HP negative n=211		HP positive n=52		p-value	OR	95%CI	
	n	%	n	%	n	%				
Gastric cancer	2	1	1	–	0	–	–	–	–	
Dysplasia	6	2	6	3	0	0.0	0.219	0.972	0.949	0.994
IM	46	17	35	17	11	21	0.438	1.349	0.632	2.879
Atrophic gastritis	50	19	41	19	9	17	0.727	0.868	0.392	1.922
Active gastritis	134	51	96	45	38	73	<0.0001	3.251	1.664	6.354
NH/FH	77	29	59	28	18	35	0.344	1.364	0.715	2.601
Gastric MALT	1	0.4	0	–	1	–	–	–	–	–

IM, intestinal metaplasia; FH, follicular hyperplasia; GI, gastrointestinal; HP, helicobacter pylori; MALT, mucosa associated lymphoid tissue; n number; NH, nodular hyperplasia; OR, odds ratio; 95%CI, 95% confidence interval. HP positivity was positively related to active gastritis only (OR 3.23, 95%CI 1.66-6.35, p<0.0001, Mann-Whitney test).

The overall prevalence of HP infection in biopsy specimens of gastric mucosa was 20% (n=52). The positivity rate of active gastritis, atrophic gastritis, and intestinal metaplasia with HP-positivity was 28%, 18%, and 24%, respectively. HP infection was significantly related to the histological feature of active gastritis (OR 3.25, 95%CI 1.66-6.35, p< 0.0001) but was not related to the presence of precancerous lesions (IM: OR 1.34, 95%CI 0.63-2.87, p=0.438; dysplasia: OR 0.97, 95%CI 0.94-0.99, p= 0.219). To note, none of the six patients with dysplasia was found to have a positive stain for HP (**Table 2**). HP was identified in 24/39 patients who had eradicated this infection before 2018 (5), showing a high recurrence rate.

Moreover, in the CVID cohort, being male (IM: OR 0.63, 95%CI 0.32-1.26, p=0.193; dysplasia: OR 0.31, 95%CI 0.03-2.67, p=0.258) and being > 50 years of age were not associated with an increased risk to develop gastric precancerous lesions (IM: OR 1.82, 95%CI 0.96-3.48; p=0.065; dysplasia: OR 2.36, 95%CI 0.42-13.09, p=0.314).

### 3.4 Gastric cancer

The GC was diagnosed in two CVID patients, already included in the previous retrospective survey.

GC was diagnosed in December 2022 in a 55-year-old male with a 30-year history of CVID. Histology showed HP-negative early gastric adenocarcinoma with surrounding areas of active gastritis, atrophic gastritis, IM, dysplasia, and nodular hyperplasia. Indication for gastroscopy was cancer gastric screening. The previous upper GI tract endoscopy, performed in April 2021, showed areas of active gastritis, atrophic gastritis and IM, HP negative. No additional risk factors for GC were identified (smoking, alcohol, obesity, history of HP infection).

The second GC was diagnosed in December 2022 in a 69-year-old female with a 57-year history of CVID. Histology showed HP-negative advanced gastric adenocarcinoma with surrounding areas of active gastritis, atrophic gastritis, IM, and dysplasia. The indication for gastroscopy was weight loss and progressive anemia. The previous upper GI tract endoscopies done in 1984, 1994, 2000, and 2012 revealed areas of intestinal metaplasia and active antral gastritis. Afterwards, the patient refused further endoscopic GC screening. Additional risk factors included a smoking habit without a history of HP infection.

**Figure 2:**
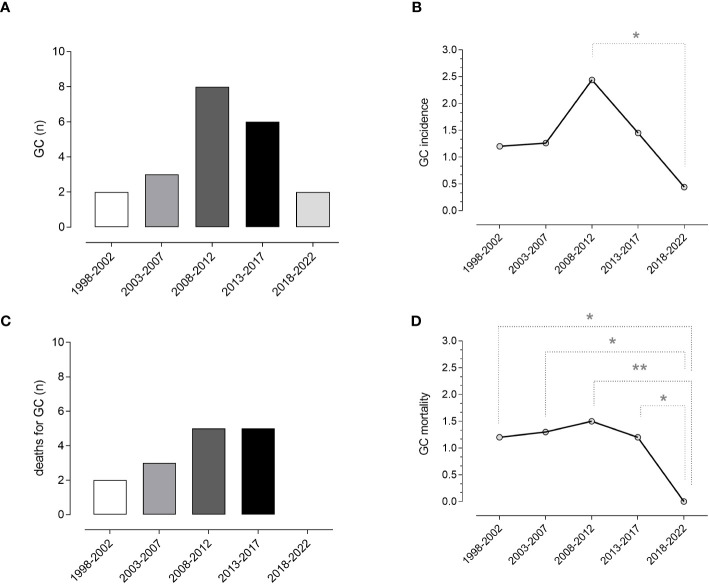
Gastric cancer and mortality. Absolute numbers **(A)** and incidence **(B)** of gastric cancer and absolute number of death for gastric cancer **(C)**, and crude mortality rate per patient **(D)** in the last 25 years (five-year intervals). Data obtained from the current and previous surveys (5) revealed a reduced incidence and crude mortality-rate for gastric cancer in CVID. Chi-square test was used to evaluate statistical significance. Two-tailed P value significances are shown as *p<0.05 and **p<0.01. GC, gastric cancer, n, number.

Overall, compared to data reported in the previous periods in our cohort (5), we recorded a lower incidence of GC. Due to low numbers, the difference is statistically significant only when compared to the period 2008-2012 (p=0.020), when we recorded the highest incidence of GC. Changes in the incidence of CG in our cohort are shown in **Figure 2**.

### 3.5 Changes over time in gastric histology

During the study time, 109 (21%) patients underwent more than one upper GI tract endoscopy (**Supplementary Table 2**). When compared to patients who underwent a single endoscopy, these ones were more likely to have precancerous lesions, such as atrophic gastritis (OR 4.38, 95%CI 2.25-8.54, p<0.0001) and IM (OR 3.27, 95%CI 1.68-6.38, p<0.0001). However, demographics and time from CVID diagnosis did not significantly differ from the rest of the cohort. Over 109 patients, 42% (n=46) were found to have precancerous lesions. Nineteen patients (41%) showed the progression from normal mucosa to precancerous lesions or from atrophic gastritis and/or IM to dysplasia or cancer. Conversely, fourteen patients (30%) with precancerous lesions showed a reversion of IM and/or atrophic gastritis to active gastritis and/or a reversion from dysplasia to IM or atrophic gastritis. The remaining 13 patients (28%) had stable precancerous abnormalities during the study time. To note, changes in histology were partially related to HP status, such as a new infection or relapse, which were documented in four patients with worsening precancerous lesions, whereas the eradication of HP was documented in one patient with downgrading of precancerous lesions.

### 3.6 Causes of death

**Figure 3:**
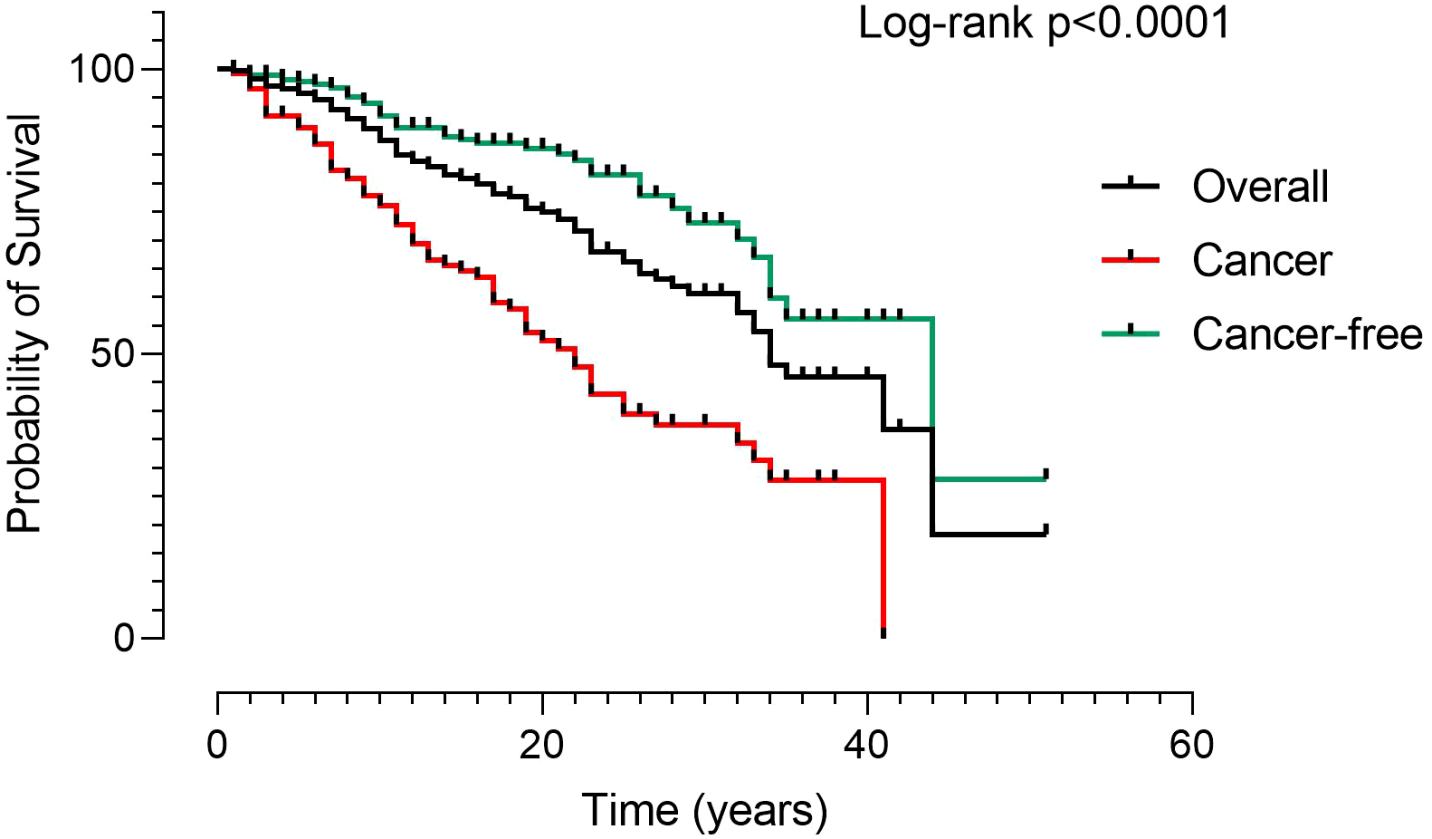
Kaplan Meier Survival curves in 512 CVID. Patients were grouped according to the presence of malignancies (black line: overall CVID population; red line: CVID with cancer; green line: CVID without cancer). The survival curves confirmed the poorer survival of patients with cancer. Log-rank test was used to evaluate statistical significance (two-tailed P value significance is indicated).

In the five-year study period, 48 deaths were recorded (**Table 3**). Infection was the main cause, accounting for 33.3% of deaths, followed by malignancies (29%). Lower respiratory tract infections leading to death from respiratory failure (19%) and death due to SARS-CoV-2 infection (8%) were separately reported. A narrow type of cancer was observed, and most deaths for malignancies were due to LNH (10%). The Kaplan-Meier survival curves confirmed (5) the poorer survival of CVID adults with cancer (Log-rank: p<0.0001) (**Figure 3**). We recorded the lack of gastric cancer as a cause of death in the prospective study (**Figure 2C**) and reduced crude mortality compared to the previous retrospective study (5) (**Figure 2D**). To note, 30/48 died patients performed an upper endoscopy within three years from the death. We can’t really exclude that the GC could not have had developed within 3 years.

**Table 3:** Causes of death were recorded in 48 CVID patients who died during the study period.

Cause of death	n=48	
	n.	%
**Infection**	16	33
LRTI (respiratory failure)	9	19
COVID-19 (respiratory failure)	4	8
Other (UTI, meningitis, sepsis)	3	6
**Cancer**	14	29
Non-Hodgkin Lymphoma	5	10
Lung cancer	3	6
Multiple myeloma	1	2
Colorectal cancer	1	2
Breast cancer	1	2
Rhinopharynx cancer	1	2
Pancreatic cancer	1	2
Liver cancer	1	2
**Cardiovascular disease**	**7**	**15**
Autoimmune manifestations*	**5**	**10**
Other*	**6**	**12**

* Autoimmune manifestations: autoimmune hemolytic anemia, idiopathic thrombocytopenic purpura, and autoimmune enteritis ** Other: Liver failure, suicide, and accident. CVID, common variable immunodeficiency; COVID-19, Coronavirus-19 disease; n, number; LRTI, low tract respiratory infections; UTI, urinary tract infection.

## 4 Discussion

In patients with Common Variable Immunodeficiency (CVID), malignancy has been reported as a main cause of death in adults (1, 4, 6–12), and in particular, patients with CVID have been widely recognized as a high-risk population for gastric cancer (5, 13, 14). In the period 2018-2022, we analyzed the prevalence of gastric cancer and precancerous lesions, by a prospective study on a cohort of 512 CVID patients. The results of this prospective study differed substantially from our previous retrospective survey (5) where we showed that GC was the first cause of death in CVID with a ten-fold excess of mortality compared to the Italian normative population. Similarly, increased incidences of GC and mortality have been confirmed by several reports across the world where a SIR was provided (7–10). We confirmed that cancers and infections remained the leading cause of death in our CVID cohort, even if no death for gastric cancer was reported. Major factors might help to clarify these conflicting results.

In the last few years, in the general population, stomach cancer predictions showed consistent mortality falls in both sexes in many countries (15–17). As shown by Collatuzzo et al. (18) analyzing time trends using the World Health Organization database, changes in age-standardized mortality rates were favorable in all countries and both sexes. Thus, our decline might go in the same direction.

A second explanation might be linked to cancer screening delays during the COVID-19 pandemic (19). In particular in Italy, compared to 2019, GC diagnosis decreased in the general population by 15.9% in 2020 (20). In fact, also in our cohort, a lower number of gastroscopies was done mainly in the year 2020 when strict lockdown measures were prescribed.

Cancer immunosurveillance plays an indispensable role in preventing GC (21), but few current practice guidelines for GC screening are available, and the appropriate timing of upper GI endoscopy in CVID is a matter of debate (13). In our CVID cohort, precancerous lesions such as dysplasia, IM and atrophic gastritis were identified in about one out of five patients, confirming data reported in other surveys (13, 22) suggesting strict monitoring due to their malignant potential (14, 23). In addition, about half of CVID patients in the current study have active gastritis, which represents itself a risk factor for developing precancerous lesions.

Gastric carcinogenesis has been widely recognized as closely related to HP (24, 25). The overall prevalence of HP infection in our study cohort from biopsy specimens of gastric mucosa was about 20%, similar to the rate found in our previous survey (5), and significantly related to active gastritis. In the CVID population, the well-known risk factors for the development of gastric cancer and precancerous gastric lesions, including diet, obesity, alcohol consumption, and smoking seem not to have a role (13).

Aggressive programs of screening and eradication of HP infection may lead to a dramatic decrease in GC mortality (18). However, in CVID, follow-up screening should not rely on HP identification only since we observed a progression to precancerous lesions also in the absence of HP. Moreover, in the current survey, CVID patients who had previously eradicated this infection showed a rate of HP recurrence accounting for 61.5%, higher than reported in the general population, now exceeding 15% (26). Contributing factors might be the underlying immune defect or antibiotic resistance due to repeated antibiotic exposure (27).

Gastric mucosa has been shown to have areas of lymphocytic gastritis and higher rates of inflammatory immune cells with alterations in a selected cytokine profile induced by HP that might impact epithelial cell biology and carcinogenesis (28, 29). Alterations in intestinal microbiota and chronic inflammatory immune response might be considered risk factors in CVID (30). Moreover, a low frequency of B-lymphocytes and plasma cells infiltrating the gastric mucosa might also contribute to reduced mucosal surveillance and gastric dysbiosis (31).

In conclusion, in the last five years, no death for GC has been recorded. We cannot rule out that the findings could be attributable to the year variability in a rare condition such as gastric cancer in CVID and to the short observation time. If this fall in mortality for GC might be real or related to underdiagnosis, it will become evident in the next few years. Moreover, this ongoing prospective study might clarify if our observations align with the GC decline recorded in the general population. In fact, despite the large number of gastroscopies analyzed, a major limitation of our study is the high number of CVID patients who did not undergo cancer gastric screening for low adherence to invasive diagnostic procedures. However, due to the high incidence of precancerous lesions, we cannot rely on the observed and predicted GC mortality trends (17, 18), and we continue to recommend tailored surveillance programs strongly.

## Data availability statement

The raw data supporting the conclusions of this article will be made available by the authors, without undue reservation.

## Ethics statement

The studies involving humans were approved by Sapienza University of Rome Ethical Commitee, Prot 316/2016 and Prot 0279/2021. The studies were conducted in accordance with the local legislation and institutional requirements. The participants provided their written informed consent to participate in this study.

## Author contributions

Conceptualization: CM, and IQ. Methodology: CM, IQ, FP. Formal analysis: FP. Investigation and data curation: GG, ES, GL, AP, CD and GC. Writing—original draft preparation: CM, IQ, and FP. Writing—review and editing: GG, FC, DF, CF, GS and SF. All authors have read and agreed to the published version of the manuscript.
