# Supplementary material for: Decline of gastric cancer mortality in common variable immunodeficiency in the years 2018-2022
Source: Front Immunol. 2023 Oct 6;14:1231242. doi: 10.3389/fimmu.2023.1231242 (PMC10587402; doi:10.3389/fimmu.2023.1231242)
Supplement: Supplementary file 1 [file Table_1.docx]

**Supplementary Material**

**Decline of gastric cancer mortality in Common Variable Immunodeficiency in the years 2018-2022**

**Cinzia Milito^1^, Federica Pulvirenti^2^ Giulia Garzi^1^, Eleonora Sculco^1^, Francesco Cinetto^3^, Davide Firinu^4^, Gianluca Lagnese^5^, Alessandra Punziano^5^, Claudia Discardi^3^, Giulia Costanzo^4^, Carla Felice^3^, Giuseppe Spadaro^5^, Simona Ferrari^6^, Isabella Quinti^1^**

^1^ Department of Molecular Medicine, Sapienza University of Rome, Rome, Italy

^2^ Reference Center for Primary Immune Deficiencies, Azienda Ospedaliera Universitaria Policlinico Umberto I, Rome, Italy.

^3^Rare Diseases Referral Center, Internal Medicine 1, Ca' Foncello Hospital, Azienda Unità Sanitaria Locale Socio-Sanitaria (AUSLSS) 2 Marca Trevigiana, Department of Medicine (DIMED), University of Padova, Padova, Italy.

^4^ Department of Medical Sciences and Public Health, University of Cagliari, Cagliari, Italy.

^5^ Department of Translational Medical Sciences, University of Naples Federico II, Naples, Italy

^6^ Medical Genetics Unit, Istituto di Ricovero e Cura a Carattere Scientifico (IRCCS) Azienda Ospedaliero-Universitaria di Bologna, 40138 Bologna, Italy.

*** Correspondence:** Isabella Quinti, isabella.quinti@uniroma1.it

**Abbreviations**

95%CI 95% confidence interval

CVID common variable immunodeficiency

COVID-19 coronavirus-19 disease

GC Gastric cancer

GI gastrointestinal

HP helicobacter pylori

IEI inborn errors of immunity

IM intestinal metaplasia

IQR interquartile range

LRTI lower tract respiratory infection

MALT mucosa associated lymphoid tissue

NH/FH nodular/follicular lymphoid hyperplasia

OR odds ratio

SIR standardized incidence ratio

UTI urinary tract infection

**Supplementary Table 1.** Characteristics of CVID patients enrolled in the retrospective study (5) and in the current prospective study.

|  | **Retrospective study (1993-2017)**  **n=455** | **Prospective study**  **(2018-2022)**  **n=512** | **p-value** |
| --- | --- | --- | --- |
| Sex (Female), n (%) | 235 (51.6) | 273 (53.7) | 0.606 |
| Age (years), median (IQR) | 51 (40-63) | 54 (41-63) | 0.078 |
| Age at CVID diagnosis, median (IQR) | 39 (28-52) | 39 (28-52) | 0.904 |
| HP prevalence in gastric histology | 40/325 (12) | 52/263 (19) | 0.016 |

**Supplementary Table 2. Upper GI tract endoscopy in 109 CVID patients who underwent more than one endoscopy during the study period.** Abbreviations: M Male F Female, HP helicobacter pylori, GC gastric cancer; PCL precancerous lesion; CD coeliac disease; IEL intra epithelial lymphocytes; FU follow up, NH nodular hyperplasia, FH follicular hyperplasia.; Act. active; Asp. aspecific; Int.Metapl. Intestinal Metaplasia

| ID | Sex | Year | Indication for EGDS | Gastric Histology | HP+ | Esophageal/ Duodenal histology | Year | Reason for to repeating | Histology | HP+ | Esophageal/ Duodenal histology | Year | Reason for repeating | Histology | HP+ | Esophageal/ Duodenal histology |
| --- | --- | --- | --- | --- | --- | --- | --- | --- | --- | --- | --- | --- | --- | --- | --- | --- |
| 87 | M | 2018 | GC screening (age, sex) | No abnormalities | Yes | Asp. villous alterations | 2021 | GC screening (no PC lesions) | Act. gastritis | No |  |  |  |  |  |  |
| 2 | M | 2018 | Chronic diarrhoea | No abnormalities | No | Asp. villous alterations | 2019 | CD clinical suspect | No abnormalities | No | Asp. villous alterations |  |  |  |  |  |
| 37 | M | 2018 | GC screening (sex, smoker) | No abnormalities | No | Asp. villous alterations | 2022 | GC screening (no PCL) | No abnormalities | No | Asp. villous alterations |  |  |  |  |  |
| 95 | F | 2018 | chronic diarrhoea | Act. gastritis | Yes | Asp. villous alterations; Giardiasis | 2019 | Chronic diarrhoea | No abnormalities | No | NH/FH | 2021 |  | Act. gastritis; Atroph. gastritis | No | NH/FH |
| 76 | M | 2018 | GE symptoms (heartburn) | No abnormalities | No | Erosive duodenitis | 2022 | GE symptoms (heartburn) | No abnormalities | No |  |  |  |  |  |  |
| 82 | F | 2018 | Esophagitis FU | No abnormalities | Yes | Erosive esophagitis | 2019 | Esophagitis FU | Act. gastritis | Yes |  |  |  |  |  |  |
| 19 | M | 2018 | GE symptoms (heartburn) | No abnormalities | No | Erosive esophagitis | 2022 | Esophagitis FU | Act. gastritis | No | Erosive esophagitis, NH/FH |  |  |  |  |  |
| 12 | M | 2018 | Esophagitis FU | No abnormalities | No | Erosive esophagitis; NH/FH | 2019 | Esophagitis FU | No abnormalities | No | Erosive esophagitis | 2021 | Esophagitis FU | No abnormalities | No | NH/FH |
| 60 | M | 2018 | chronic diarrhoea | Act. gastritis | No | Giardiasis | 2021 | Screening CG (no PC lesions) | Act. gastritis | No |  |  |  |  |  |  |
| 88 | M | 2018 | GE symptoms (heartburn) | Act. gastritis | No | Esophageal squamous papilloma | 2019 | Esophageal squamous papilloma FU | Act. gastritis | Yes | NH/FH | 2020 | GE symptoms | Act. gastritis | Yes | NH/FH |
|  |  |  |  |  |  |  | 2021 | GE symptoms | Act. gastritis, MALT | Yes | NH/FH | 2021 | Gastric MALT | Act. gastritis; Atroph. gastritis; | Yes | NH/FH |
| 1 | M | 2018 | CD clinical suspect | No abnormalities | No | IEL 25/100 | 2020 | Atroph. enteritis refractory to GFD | No abnormalities | No | IEI >25/100 | 2021 | Atroph. enteritis refractory to GFD | Act. gastritis; Atroph. gastritis | No | IEI >25/100; |
| 32 | F | 2018 | CD FU in GFD | Act. gastritis | No | IEL> 25/100; NH/FH | 2021 | CD FU in GFD | No abnormalities | No | IEI >25/100 |  |  |  |  |  |
| 89 | M | 2018 | CD FU in GFD | Act. gastritis | Yes | IEL>25/100 | 2021 | CD FU in GFD | Act. gastritis | No | IEL>25/100 | 2022 | CD refractory to GFD | Act. gastritis | Yes | IEL>25/100 |
| 92 | M | 2018 | HP colonization | Act. Gastritis; Atroph. gastritis; Int.metapl.; | Yes | NH/FH | 2019 | GC screening | Atroph. gastritis | No | NH/FH | 2022 |  | Act. gastritis; Atroph. gastritis; Int. metapl. | No |  |
| 90 | F | 2018 | Portal hypertension | No abnormalities | No | NH/FH | 2022 | Portal hypertension | Act. gastritis | Yes | NH/FH |  |  |  |  |  |
| 48 | F | 2018 | Previous finding of histological alterations | Act. gastritis; Atroph. gastritis; Int.metapl. | No | NH/FH | 2019 | GC screening | Act. gastritis; Atroph. gastritis; Int.metapl. | No | IEI >25/100; NH/FH; Giardiasis |  |  |  |  |  |
| 71 | F | 2018 | CD FU | Act. gastritis | No | NH/FH | 2021 | CD FU | Atroph. gastritis | No | IEL>25/100 |  |  |  |  |  |
| 103 | F | 2018 | chronic diarrhoea | Act. gastritis | Yes | NH/FH | 2021 | chronic diarrhoea | No abnormalities | Yes |  |  |  |  |  |  |
| 4 | M | 2018 | Previous finding of histological alterations | Act. gastritis; Erosive gastritis; Int. metapl. | No |  | 2019 | GC screening | Act. gastritis; Atroph. gastritis; Int. metapl. | No |  | 2020 | GC screening | Act. gastritis; Atroph. gastritis; Int. metapl.; Dysplasia | No |  |
|  |  |  |  |  |  |  | 2021 | GC screening | Act. gastritis; Atroph. gastritis; Int. metapl. | No |  | 2022 | GC screening | Act. gastritis; Atroph. gastritis; Int. metapl. | No |  |
| 96 | M | 2018 | chronic diarrhoea | Act. gastritis | Yes | NH/FH; Giardiasis | 2019 | chronic diarrhoea | No abnormalities | No |  |  |  |  |  |  |
| 98 | F | 2018 | HP colonization | Act. Gastritis | Yes |  | 2019 | GC screening (no PC lesions) | Act. gastritis; Atroph. gastritis; Int.metapl. | Yes |  | 2022 | GC screening | Act. gastritis; Atroph. gastritis; Int. metapl. | Yes |  |
|  |  |  |  |  |  |  | 2022 | GC screening | Act. gastritis; Atroph. gastritis; Int.metapl. | Yes |  |  |  |  |  |  |
| 101 | M | 2018 | Previous finding of histological alterations | Act. gastritis; Int.metapl. | Yes |  | 2019 | GC screening | Act. gastritis; Int.metapl. | Yes |  | 2022 | GC screening | Act. gastritis; Int.metapl. | No |  |
| 85 | M | 2018 |  | No abnormalities | No |  | 2019 | CD clinical suspect | Act. gastritis | Yes | IEI >25/100; NH/FH; | 2020 | CD FU after GFD | Act. gastritis | No |  |
| 66 | M | 2018 | chronic diarrhoea | Act. gastritis; Atroph. gastritis; Int.metapl. | No |  | 2020 | GC screening | Int.metapl. | No |  | 2021 | GC screening | Act. gastritis; Int.metapl. | No |  |
| 108 | F | 2018 | Previous finding of histological alterations | Act. gastritis; Atroph. gastritis; Intestinal metapl. | No |  | 2020 | GC screening | Act. gastritis; Int.metapl. | Yes |  | 2022 | GC screening | Int.metapl. | Yes | NH/FH |
| 40 | M | 2018 | Previous finding of histological alterations | No abnormalities | No |  | 2019 | GC screening (no PC lesions) | Gastric hyperplastic polyp | No | NH/FH | 2022 |  | No abnormalities |  |  |
| 91 | M | 2018 | GE symptoms | No abnormalities | No |  | 2019 | CD FU in GFD | No abnormalities | Yes |  | 2022 |  | No abnormalities | No |  |
| 63 | M | 2018 | CD FU in GFD | No abnormalities | No |  | 2019 | Screening CG (no PC lesions) | No abnormalities | No |  | 2022 |  | No abnormalities |  |  |
| 13 | M | 2018 | GE symptoms | No abnormalities | No |  | 2021 | GC screening (no PC lesion) | Act. gastritis | No | NH/FH |  |  |  |  |  |
| 5 | F | 2018 | GC screening (sex, smoker) | Act. gastritis | No |  | 2019 | Portal hypertension | Act. gastritis | No | NH/FH |  |  |  |  |  |
| 58 | M | 2018 | Portal hypertension | Act. gastritis; Atroph. gastritis | No |  | 2021 | Portal hypertension | Act. gastritis | No | Asp. villous alterations |  |  |  |  |  |
| 10 | M | 2018 | Portal hypertension | Act. gastritis | No |  | 2020 | Screening CG (no PC lesions) | Act. gastritis | No |  |  |  |  |  |  |
| 100 | M | 2018 | GC screening (sex) | Act. Gastritis; Atroph. gastritis; Intestinal metapl. | Yes |  | 2019 | GC screening | Act. gastritis; Atroph. gastritis | Yes | NH/FH |  |  |  |  |  |
| 16 | F | 2018 | Previous finding of histological alterations | Act. gastritis | No |  | 2022 | GC screening (no PC lesions) | Act. gastritis; Atroph. gastritis | No |  |  |  |  |  |  |
| 43 | F | 2018 | Previous finding of histological alterations | Act. gastritis | No |  | 2021 | GC screening (no PC lesions) | Atroph. gastritis | No |  |  |  |  |  |  |
| 21 | F | 2018 | GC screening (age, smoker) | No abnormalities | No |  | 2021 | GC screening (no PC lesions) | Atroph. gastritis | No | NH/FH |  |  |  |  |  |
| 93 | M | 2018 | GC screening (age, high BMI) | No abnormalities | No |  | 2019 | chronic diarrhoea | No abnormalities | Yes | NH/FH; Asp. villous alterations |  |  |  |  |  |
| 86 | M | 2018 | chronic diarrhoea | Act. gastritis; Atroph. gastritis; Int.metapl.; Dysplasia | No |  | 2019 | GC screening | No abnormalities | Yes | NH/FH |  |  |  |  |  |
| 14 | F | 2018 | Previous finding of histological alterations | No abnormalities | No |  | 2022 | GC screening (no PC lesion) | No abnormalities | No |  |  |  |  |  |  |
| 20 | M | 2018 | GC screening (no RF) | Act. gastritis | No |  | 2022 | GC screening (no PC lesions) | No abnormalities | No |  |  |  |  |  |  |
| 31 | F | 2018 | GE symptoms | Act. gastritis | No |  | 2022 | GC screening (no PC lesions) | No abnormalities | No |  |  |  |  |  |  |
| 30 | M | 2018 | GE symptoms | No abnormalities | No |  | 2022 | GC screening (no PC lesions) | No abnormalities | No |  |  |  |  |  |  |
| 51 | M | 2018 | GC screening (sex/no RF) | No abnormalities | No |  | 2019 | GC screening (no PC lesions) | No abnormalities | No |  |  |  |  |  |  |
| 77 | M | 2018 | GC screening (age, sex) | No abnormalities | No |  | 2019 | GC screening (no PC lesions) | No abnormalities | No |  |  |  |  |  |  |
| 70 | M | 2018 | GC screening (sex/no RF) | No abnormalities | No |  | 2022 | GC screening (no PCL) | No abnormalities | No |  |  |  |  |  |  |
| 27 | M | 2018 | GC screening (sex/no RF) | Act. gastritis | No |  | 2020 | Screening CG (no PC lesions) | No abnormalities | No | NH/FH |  |  |  |  |  |
| 74 | F | 2018 | GC screening  (age, sex) | Act. gastritis | No |  | 2020 | Screening CG (no PC lesions) | No abnormalities | No |  |  |  |  |  |  |
| 6 | F | 2018 | GC screening (no RF) | No abnormalities | No |  | 2019 | Screening CG (no PC lesions) | No abnormalities | No |  |  |  |  |  |  |
| 57 | M | 2018 | GC screening (no RF) | No abnormalities | No |  | 2019 | Screening CG (no PC lesions) | No abnormalities | No | Asp. villous alterations |  |  |  |  |  |
| 15 | M | 2018 | chronic diarrhoea | Act. gastritis | No |  | 2021 | Screening CG (no PC lesions) | No abnormalities | No |  |  |  |  |  |  |
| 8 | M | 2019 | GE symptoms | No abnormalities | No | Asp. villous alterations | 2021 | CD clinical suspect | No abnormalities | No | Asp. villous alterations | 2022 | CD clinical suspect | No abnormalities | No | Asp. villous alterations |
| 3 | M | 2019 | chronic diarrhoea | No abnormalities | No | Asp. villous alterations | 2022 | CD clinical suspect | No abnormalities | No |  |  |  |  |  |  |
| 41 | F | 2019 | chronic diarrhoea | No abnormalities | No | Asp. villous alterations; NH/FH; Giardiasis | 2020 | chronic diarrhoea | No abnormalities | No |  |  |  |  |  |  |
| 104 | M | 2019 | chronic diarrhoea | Act. gastritis | Yes | Erosive esophagitis; NH/FH | 2021 | Erosive esophagitis FU | Act. gastritis | No | NH/FH |  |  |  |  |  |
| 59 | F | 2019 | GE symptoms | No abnormalities | No | NH/FH | 2022 | Screening CG (no PC lesions) | Act. gastritis | No |  |  |  |  |  |  |
| 99 | M | 2019 | GE symptoms (heartburn) | Act. gastritis; Atroph. gastritis | Yes | NH/FH | 2021 | Portal Hypertension | Act. gastritis; Atroph. gastritis | No | NH/FH |  |  |  |  |  |
| 50 | M | 2019 | Portal Hypertension | No abnormalities | No | NH/FH | 2021 | Screening CG (no PC lesions) | Act. gastritis; Atroph. gastritis | No | Giardiasis |  |  |  |  |  |
| 22 | M | 2019 | GC screening (sex, previous HP) | No abnormalities | No | NH/FH | 2022 | GC screening (no PC lesions) | No abnormalities | No | Erosive esophagitis; NH/FH |  |  | 22 | M | 2019 |
| 83 | F | 2019 | GC screening (age, sex, high BMI) | Int.metapl. | No | NH/FH | 2021 | GC screening | No abnormalities | Yes |  |  |  |  |  |  |
| 67 | M | 2019 | Previous finding of histological alterations, HP colonization | Act. gastritis | No | NH/FH, Duodenal polyposis | 2022 | Duodenal polyposis FU | No abnormalities | No | NH/FH |  |  |  |  |  |
| 107 | M | 2019 | GC screening (sex/no RF) | Act. gastritis | Yes | NH/FH | 2022 | GC screening (no PC lesions) | Act. gastritis | Yes |  |  |  |  |  |  |
| 33 | F | 2019 | HP colonization | No abnormalities | No |  | 2021 | Screening CG (no PC lesions) | Act. gastritis | No |  | 2022 |  | No abnormalities |  |  |
| 68 | F | 2019 | GC screening (age) | Atroph. gastritis | No |  | 2022 | Esophagitis FU | Act. gastritis | No | NH/FH |  |  |  |  |  |
| 38 | F | 2019 | GE symptoms | Act. gastritis; Atroph. gastritis; Int. metapl. | No |  | 2022 | GC screening | Act. gastritis | No |  |  |  |  |  |  |
| 65 | F | 2019 | Previous finding of histological alterations | Act. gastritis; Atroph. gastritis; Int.metapl. | No |  | 2022 | GC screening | Act. gastritis | No |  |  |  |  |  |  |
| 79 | M | 2019 | Previous finding of histological alterations | No abnormalities | Yes |  | 2020 | Portal hypertension | Act. gastritis; Atroph. gastritis | Yes |  |  |  |  |  |  |
| 18 | M | 2019 | Portal hypertension | Atroph. gastritis; Int.metapl. | No |  | 2021 | GC screening | Act. gastritis; Atroph. gastritis; Int.metapl. | No | IEI >25/100 |  |  |  |  |  |
| 45 | F | 2019 | Previous finding of histological alterations | No abnormalities | No |  | 2022 | GC screening (no PC lesions) | Act. gastritis; Gastric hyperplastic polyp | No | NH/FH |  |  |  |  |  |
| 84 | M | 2019 | GE symptoms | No abnormalities | Yes |  | 2022 | GC screening (no PC lesions) | Act. gastritis; Int.metapl. | No | NH/FH |  |  |  |  |  |
| 80 | M | 2019 | GC screening (age, sex) | Act. gastritis; Int.metapl.; Dysplasia | Yes |  | 2020 | GC screening | Act. gastritis; Int.metapl.; Dysplasia | Yes |  |  |  |  |  |  |
| 102 | F | 2019 | Previous finding of histological alterations | Act. gastritis; Atroph. gastritis | Yes |  | 2020 | chronic diarrhoea | Atroph. gastritis | Yes |  |  |  |  |  |  |
| 35 | M | 2019 | chronic diarrhoea | No abnormalities | No |  | 2022 | Chronic diarrhoea | Int.metapl. | No |  |  |  |  |  |  |
| 62 | M | 2019 | Chronic diarrhoea | No abnormalities | No |  | 2022 | Screening CG (no PC lesions) | Int.metapl. | No | NH/FH |  |  |  |  |  |
| 53 | M | 2019 | GE symptoms (heartburn) | Act. gastritis | No |  | 2022 | Esophagitis FU | No abnormalities | No | NH/FH |  |  |  |  |  |
| 46 | F | 2019 | Esophagitis FU | Act. gastritis; Atroph. gastritis; Int.metapl. | No |  | 2020 | GC screening | No abnormalities | No |  |  |  |  |  |  |
| 109 | M | 2019 | Previous finding of histological alterations | Atroph. gastritis | Yes |  | 2022 | GC screening | No abnormalities | No |  |  |  |  |  |  |
| 44 | M | 2019 | Previous finding of histological alterations | Int.metapl. | No |  | 2022 | GC screening | No abnormalities | No |  |  |  |  |  |  |
| 9 | F | 2019 | Previous finding of histological alterations | No abnormalities | No |  | 2021 | GC screening (no PC lesion) | No abnormalities | No | NH/FH |  |  |  |  |  |
| 49 | M | 2019 | GC screening (age, smoker) | No abnormalities | No |  | 2020 | GC screening (no PC lesions) | No abnormalities | No |  |  |  |  |  |  |
| 17 | M | 2019 | GC screening (age, sex) | Int.metapl.; Act. gastritis | No |  | 2022 | Portal hypertension | No abnormalities | No |  |  |  |  |  |  |
| 29 | M | 2019 | Portal hypertension | No abnormalities | No |  | 2021 | Portal hypertension | No abnormalities | No |  |  |  |  |  |  |
| 81 | F | 2019 | Portal hypertension | Act. gastritis | No |  | 2022 | Screening CG (no PC lesions) | No abnormalities | Yes | NH/FH |  |  |  |  |  |
| 24 | M | 2019 | GC screening (no RF) | Act. gastritis | No |  | 2022 | Screening CG (no PC lesions) | No abnormalities | No |  |  |  |  |  |  |
| 34 | M | 2019 | GE symptoms | No abnormalities | No |  | 2021 | Screening CG (no PC lesions) | No abnormalities | No | NH/FH |  |  |  |  |  |
| 56 | M | 2019 | GC screening (sex/no RF) | No abnormalities | No |  | 2022 | Screening CG (no PC lesions) | No abnormalities | No |  |  |  |  |  |  |
| 61 | M | 2019 | GC screening (sex/no RF) | No abnormalities | No |  | 2022 | Screening CG (no PC lesions) | No abnormalities | No |  |  |  |  |  |  |
| 73 | M | 2019 | GC screening (age, sex) | No abnormalities | No |  | 2022 | Screening CG (no PC lesions) | No abnormalities | No |  |  |  |  |  |  |
| 78 | F | 2019 | GC screening (age, sex) | No abnormalities | No |  | 2022 | Screening CG (no PC lesions) | No abnormalities | No | NH/FH |  |  |  |  |  |
| 26 | M | 2020 | GC screening (age) | No abnormalities | No | Asp. villous alterations | 2022 | Screening CG (no PC lesions) | No abnormalities | No | Duodenal polyposis |  |  |  |  |  |
| 28 | M | 2020 | GC screening (sex/no RF) | Act. Gastritis; Atroph. gastritis; Int.metapl. | No | Barrett esophagus; NH/FH | 2022 | GC screening | Act. gastritis; Int.metapl. | No |  |  |  |  |  |  |
| 106 | F | 2020 | Previous finding of histological alterations | Act. gastritis | Yes |  | 2022 | GC screening (no PC lesions) | Act. gastritis | Yes |  |  |  |  |  |  |
| 7 | M | 2020 | GE symptoms | No abnormalities | No |  | 2022 | Screening CG (no PC lesions) | Act. gastritis | No | NH/FH |  |  |  |  |  |
| 94 | F | 2020 | GC screening (age, sex, smoker) | Act. Gastritis; Atroph. gastritis; Int.metapl | No |  | 2021 | GC screening | Act. gastritis; Int.metapl. | Yes |  |  |  |  |  |  |
| 69 | M | 2020 | Previous finding of histological alterations | No abnormalities | No |  | 2022 | Screening CG (no PC lesions) | Act. gastritis; Int.metapl. | No |  |  |  |  |  |  |
| 105 | F | 2020 | GC screening (age, sex) | Act. gastritis | Yes |  | 2022 | GC screening (no PC lesions) | Atroph. gastritis | No |  |  |  |  |  |  |
| 64 | F | 2020 | GE symptoms | No abnormalities | No |  | 2021 | Portal hypertension | Atroph. gastritis | No |  |  |  |  |  |  |
| 55 | F | 2020 | Portal hypertension | Act. gastritis; Atroph. gastritis; Int.metapl. | No |  | 2021 | GC screening | Int.metapl. | No | NH/FH |  |  |  |  |  |
| 36 | F | 2020 | Previous finding of histological alterations | Int.metapl. | No |  | 2021 | GC screening | No abnormalities | No |  |  |  |  |  |  |
| 25 | F | 2020 | Previous finding of histological alterations | No abnormalities | No |  | 2021 | GC screening (no PC lesions) | No abnormalities | No |  |  |  |  |  |  |
| 39 | M | 2020 | GC screening (age) | No abnormalities | No |  | 2022 | Screening CG (no PC lesions) | No abnormalities | No |  |  |  |  |  |  |
| 52 | F | 2021 | GC screening (sex/no RF) | Atroph. gastritis | No | Asp. villous alterations; IEL>25/100 | 2022 | CD clinical suspect | Atroph. gastritis | No | IEI >25/100 |  |  |  |  |  |
| 42 | M | 2021 | CD clinical suspect | Act. gastritis; Atroph. gastritis; Int.metapl.; Dysplasia | No | IEL >25/100; NH/FH | 2022 | GC screening | Act. gastritis; Atroph. gastritis; Int.metapl. | No | IEI >25/100; NH/FH |  |  |  |  |  |
| 97 | M | 2021 | GE symptoms | No abnormalities | Yes | NH/FH | 2022 | GE symptoms | Act. gastritis | No |  |  |  |  |  |  |
| 75 | M | 2021 | GE symptoms | Act. Gastritis; Atroph. gastritis; Int. metapl. | No | NH/FH | 2022 | GC screening | Act. gastritis, Atroph. gastritis; Int.metapl; Dysplasia; intramucosal adenocarcinoma | No | NH/FH |  |  |  |  |  |
| 72 | M | 2021 | Previous finding of histological alterations | Int.metapl. | No | NH/FH | 2022 | GC screening/ CD FU in GFD | No abnormalities | No | IEI >25/100 |  |  |  |  |  |
| 11 | M | 2021 | CD FU in GFD | No abnormalities | No | NH/FH | 2022 | GC screening (no PC lesions) | No abnormalities | No |  |  |  |  |  |  |
| 54 | M | 2021 | GC screening (sex/no RF) | Act. gastritis | No | NH/FH; Giardiasis | 2022 | chronic diarrhoea | No abnormalities | No | NH/FH; Giardiasis |  |  |  |  |  |
| 47 | F | 2021 | chronic diarrhoea | Act. gastritis; Atroph. gastritis; Int.metapl. | No |  | 2022 | GC screening | Act. gastritis; Atroph. gastritis; Int.metapl. | No |  |  |  |  |  |  |
| 23 | M | 2021 | Previous finding of histological alterations | No abnormalities | No |  | 2022 | GC screening (no PC lesions) | No abnormalities | No | Erosive esophagitis; NH/FH |  |  |  |  |  |
|  |  |  |  |  |  |  | 2022 | GC screening | Act. gastritis; Int.metapl. | Yes |  |  |  |  |  |  |
